# Microglia and monocyte-derived macrophages: functionally distinct populations that act in concert in CNS plasticity and repair

**DOI:** 10.3389/fncel.2013.00034

**Published:** 2013-04-08

**Authors:** Anat London, Merav Cohen, Michal Schwartz

**Affiliations:** Department of Neurobiology, Weizmann Institute of ScienceRehovot, Israel

**Keywords:** microglia, monocytes, CNS, innate, resolution of inflammation, macrophages, neuroprotection, monocyte-derived macrophages

## Abstract

Functional macrophage heterogeneity is recognized outside the central nervous system (CNS), where alternatively activated macrophages can perform immune-resolving functions. Such functional heterogeneity was largely ignored in the CNS, with respect to the resident microglia and the myeloid-derived cells recruited from the blood following injury or disease, previously defined as blood-derived microglia; both were indistinguishably perceived detrimental. Our studies have led us to view the myeloid-derived infiltrating cells as functionally distinct from the resident microglia, and accordingly, to name them monocyte-derived macrophages (mo-MΦ). Although microglia perform various maintenance and protective roles, under certain conditions when they can no longer provide protection, mo-MΦ are recruited to the damaged CNS; there, they act not as microglial replacements but rather assistant cells, providing activities that cannot be timely performed by the resident cells. Here, we focus on the functional heterogeneity of microglia/mo-MΦ, emphasizing that, as opposed to the mo-MΦ, microglia often fail to timely acquire the phenotype essential for CNS repair.

## INTRODUCTION

Outside the central nervous system (CNS), macrophages are known to acquire distinct phenotypes, and accordingly, perform various different and even opposing functions. Macrophages are generally polarized into two major phenotypes: Th1-related cytokines, such as interferon-gamma (IFN-γ), and microbial challenge by products such as lipopolysaccharides (LPS) induce the classically activated M1 phenotype, driving macrophages toward a pro-inflammatory microbicidal function, whereas Th2 cytokines, such as interleukin-4 (IL-4) and IL-13 polarize macrophages to an alternatively activated M2 phenotype associated with wound healing and immune resolution (Gordon, 2003; Gordon and Taylor, 2005; Mantovani et al., 2005; Mosser and Edwards, 2008; Auffray et al., 2009; Martinez et al., 2009; Gordon and Martinez, 2010; Sica and Mantovani, 2012). These different macrophage populations act following various insults outside the CNS where the CBR2^+^CX_3_BR1^low^Ly6C^high^ subset, corresponding to the M1 phenotype, is the first recruited to the damage site and is typically pro-inflammatory, while the CBR2^-^CX_3_BR1^high^Ly6C^low^ cells, matching the M2 phenotype, subsequently terminate the local inflammation as well as promoting regeneration and healing (Arnold et al., 2007; Nahrendorf et al., 2007). These two polarized phenotypes are further classified, based on their diverse surface markers, phenotypes and functions, into a more continuum spectrum of macrophage repertoire (Mosser and Edwards, 2008).

In the CNS, however, such diversity of macrophage functions was largely overlooked, as microglia, the native immune cells of the CNS, were considered its exclusive innate components. Initially discovered by Cajal (1913) and his student Del Rio-Hortega (1919), microglia are currently accepted as self-renewing cells with a unique embryonic origin (Ginhoux et al., 2010; Schulz et al., 2012; Gomez Perdiguero et al., 2013), distributed along CNS parenchymal tissues. Their primary roles are the maintenance of normal CNS functions (Elkabes et al., 1996; Nakajima et al., 2001; Aarum et al., 2003; Nimmerjahn et al., 2005; Walton et al., 2006; Ziv et al., 2006; Ransohoff and Perry, 2009; Wake et al., 2009; Sierra et al., 2010; Tremblay, 2011) and the continuous search for alterations in homeostasis through their constantly scanning dynamic ramifications (Nimmerjahn et al., 2005; Hanisch and Kettenmann, 2007; Ransohoff and Perry, 2009; Kettenmann et al., 2011). Under pathological conditions, microglial functions are largely dependent on their activation stimuli; whereas moderate CNS damage evokes protection by microglia (Prewitt et al., 1997; Rabchevsky and Streit, 1997; Batchelor et al., 1999; Chung et al., 1999; Butovsky et al., 2001; Kotter et al., 2001; Stadelmann et al., 2002; Streit, 2002; Shaked et al., 2004, 2005; Neumann et al., 2006, 2008; Schwartz et al., 2006; Yin et al., 2006; Majumdar et al., 2007; Muzio et al., 2007; Thored et al., 2009; Kettenmann et al., 2011), intensive acute activation (for example in spinal cord injury, optic nerve crush, or stroke) and chronic activation, which characterizes neurodegenerative diseases, render these cells neurotoxic, potentially impairing neuronal activity (Munn, 2000; Stalder et al., 2001; Wegiel et al., 2001; Ekdahl et al., 2003; Monje et al., 2003; Stirling et al., 2004; Block and Hong, 2005; Heppner et al., 2005; Block et al., 2007; Hanisch and Kettenmann, 2007; Muzio et al., 2007; Ovanesov et al., 2008; Centonze et al., 2009; Maezawa and Jin, 2010; Perry et al., 2010; ; Scheffel et al., 2012). Such a phenotype not only prohibits microglia from resolving inflammatory damage but rather contributes to the vicious cycle of toxicity and calls for additional assistance to terminate the local inflammation.

As an immune privileged site, the CNS was, for decades, considered sealed for leukocyte entry, protected from the circulation behind the walls of the blood–brain barrier (Wilson et al., 2010; Ransohoff and Engelhardt, 2012). Any recruitment of immune cells to the CNS was perceived as either a technical artifact (Ajami et al., 2007; Mildner et al., 2007) or as part of the ongoing inflammatory damage (McGeer et al., 1990; Popovich et al., 1999; Gris et al., 2004; Stirling et al., 2004; Boster et al., 2008). Further confusion resulted from the fact that blood-derived myeloid cells, recruited following CNS damage, were considered microglial reinforcements of comparable functions, and were accordingly termed “blood-derived microglia” (Eglitis and Mezey, 1997; Priller et al., 2001; Bechmann et al., 2005; Simard et al., 2006). A series of recent innovative studies demonstrated that such infiltrating cells, which we defined as monocyte-derived macrophages (mo-MΦ), perform indispensable roles that cannot be provided by their resident counterparts (Shechter et al., 2009, 2011; London et al., 2011). These studies challenged the traditional perception of macrophages in the CNS as a functionally homogeneous population. Moreover, they set the ground for a new era of research employing sophisticated techniques including parabiosis, head-protected bone marrow chimeras, transgenic mice, and fate mapping analysis (Carson et al., 2007; Ginhoux et al., 2010; Saederup et al., 2010; Ajami et al., 2011; Mildner et al., 2011; Prinz et al., 2011; Butovsky et al., 2012; Derecki et al., 2012; Schulz et al., 2012; Colton, 2013; Gomez Perdiguero et al., 2013), aimed at revealing the differential origin, phenotype, and function of distinct myeloid populations within the CNS. In this perspective, we will focus on the functional heterogeneity of microglia/mo-MΦ, addressing microglial functions as the first immunological support, the failure of these cells to provide significant protection under intensive acute or chronic activation, and the subsequent unique contribution of the mo-MΦ.

## MICROGLIA IN MAINTENANCE AND DEFENSE

Similar to other tissue-resident macrophages outside the CNS, the primary role of microglia is to support normal tissue function, in this case neuronal integrity (Nimmerjahn et al., 2005; Hanisch and Kettenmann, 2007; Ransohoff and Perry, 2009; Kettenmann et al., 2011; Scheffel et al., 2012). The development of *in vivo* two-photon microscopy revolutionized our understanding of microglial functions under steady state. It allowed the study of non-activated microglia in intact brains of living animals (Davalos et al., 2005; Hanisch and Kettenmann, 2007). In these studies, using *Cx3BR1^GFP/+^* or Iba1-EGFP transgenic mice, in which microglia are fluorescently labeled (Jung et al., 2000; Hirasawa et al., 2005), these cells present highly motile processes, which directly contact astrocytes, neurons, and blood vessels, allowing the microglia to perform surveillance functions, constantly sensing subtle changes in their microenvironment (Nimmerjahn et al., 2005). Microglia provide several housekeeping functions. For instance, these cells are involved in the maintenance of synapses; microglial ramifications directly interact with termini, spines, astrocytic processes, and synaptic clefts (Murabe and Sano, 1982; Wake et al., 2009; Tremblay, 2011). These interactions enable the recognition of neuronal activity or structural alterations, according to which microglia facilitate synapse elimination, pruning or maturation, thereby preserve and organize neuronal networks (Murabe and Sano, 1982; Wake et al., 2009; Paolicelli et al., 2011; Tremblay, 2011). Microglia have been reported to support neurogenesis; they rapidly and efficiently clear out, by phagocytosis, the numerous apoptotic neural progenitor cells (NPCs) that do not incorporate into the circuitry (Sierra et al., 2010), direct the migration and differentiation of NPCs, as well as secreting soluble factors promoting neurogenesis (Aarum et al., 2003; Butovsky et al., 2006c; Walton et al., 2006; Choi et al., 2008). Moreover, microglial activation following exercise and by local interaction with adaptive immune cells strongly supports neurogenesis and enhances cognitive functions (Ziv et al., 2006; Ziv and Schwartz, 2008; Wolf et al., 2009; Vukovic et al., 2012). Additionally, several *in vitro* and *in vivo* studies demonstrated the capacity of microglia to secrete neurotrophic factors, e.g., nerve growth factor (NGF), neurotrophin-3 (NT-3), and NT-4 (Elkabes et al., 1996; Nakajima et al., 2001). Under certain conditions microglia upregulate their brain-derived neurotrophic factor (BDNF) and insulin-like growth factor-1 (IGF-1) expression; both factors have protective and growth-promoting effects and are essential for learning and memory skills (Mizuno et al., 2000; Hsieh et al., 2004; Lee et al., 2004; Butovsky et al., 2006c; Wang et al., 2012).

Being the native immune cells of the CNS, microglia act as the first line of defense, protecting the CNS from invading agents as well as internal enemies; microglia are involved in infection, inflammation, autoimmune disease, trauma, ischemia, and neurodegeneration. After initial exposure to a danger signal, microglia become activated; they upregulate expression levels of certain molecules such as CD11b and Iba1, and gain expression of molecules associated with antigen presentation, such as major histocompatibility complex (MHC)-II, B7.1, and B7.2 (CD80/86), which are absent in naïve microglia. Microglia then lose their ramified morphology and surveillance mode, and convert to amoeboid-like, functional cells (Kettenmann et al., 2011).

Microglial functions under pathological conditions may reflect their diverse phenotypes acquired contingent to their activation signals. For example, activation of microglia by T cells that recognize CNS antigens or T cell-derived cytokines such as IFN-γ (at low concentrations) and IL-4 supports differentiation of NPCs and provides neuroprotection by regulating IGF-1 and tumor necrosis factor-alpha (TNF-α) levels. However, stimulation with LPS, amyloid-β or high concentrations of IFN-γ diminishes these effects. Moreover, activation of microglia by IL-4 prior to the LPS stimulation prevents the LPS-mediated-inhibition of the microglial neuroprotective effects (Avidan et al., 2004; Shaked et al., 2004; Butovsky et al., 2005, 2006a,b,c; Scheffel et al., 2012). Thus, microglia are highly versatile cells; their regulated activation and proper termination might help in tissue preservation, repair, and renewal, while intensive acute or chronic activation may result in irreversible tissue loss.

Microglia exert several protective roles. These include removal by phagocytosis of pathogens and microbes, as well as clearance of toxic molecules, cell debris, remains of extracellular matrix, myelin derivatives, and protein deposits (e.g., amyloid-β or p-tau), all of which further contribute to the local inflammation and are inhibitory to regeneration and repair (Chung et al., 1999; Magnus et al., 2001; Ravichandran, 2003; Shaked et al., 2005; Liu et al., 2006; Majumdar et al., 2007; Kettenmann et al., 2011). Microglia can promote regeneration; other than removal of growth-inhibitory compounds (Kettenmann et al., 2011), these cells produce classical growth factors required for remyelination and regeneration (Kotter et al., 2001; Stadelmann et al., 2002). Microglia were reported to support regeneration of the optic nerve as well as sensory axons in the injured spinal cord (Prewitt et al., 1997; Rabchevsky and Streit, 1997; Yin et al., 2006) and to induce dopaminergic sprouting in the injured striatum (Batchelor et al., 1999). Additionally, microglia were shown to support neurogenesis and reduce neuronal death following stroke (Neumann et al., 2006; Thored et al., 2009).

## MICROGLIAL MALFUNCTION FOLLOWING INTENSIVE ACUTE OR CHRONIC ACTIVATION – A CALL FOR PERIPHERAL HELP

Substantial evident suggest that microglial activity is not always optimal and is often not sufficient to support significant CNS repair; on the contrary in many cases their insufficient support turns detrimental. Under intensive acute or chronic activation, microglia not only fail to provide the needed functions, but there are ample evidence implying that they can be actively deleterious; these cells secrete reactive oxygen species, nitric oxide (NO), and pro-inflammatory cytokines that can endanger neurons, oligodendrocytes, or essential structures of the extracellular matrix (Monje et al., 2003; Stirling et al., 2004; Block and Hong, 2005; Block et al., 2007; Hanisch and Kettenmann, 2007; Perry et al., 2010). Microglial malfunction was suggested as a possible etiology in schizophrenia, resulting in impaired pruning during neurodevelopment, disturbance of normal neurotransmitter function, and uncontrolled production of pro-inflammatory cytokines such as TNF and IL-6, as well as failure in clearance of neuronal corpses (Munn, 2000). Microglial abnormal response is also evident in Rett syndrome, a neurodevelopmental disease resulting from mutation of the gene encoding methyl-CpG binding protein (*Mecp2*). *Mecp2*-null microglia release glutamate at neurotoxic levels (Maezawa and Jin, 2010) and have impaired phagocytic activity (Derecki et al., 2012, 2013), possibly contributing to disease development. Impaired microglial function is also reported in amyotrophic lateral sclerosis (ALS), where microglia derived from mutant superoxide dismutase 1 (SOD1) mouse, an established disease model of ALS, induce more oxidative stress and cause higher neuronal loss compared with wt microglia (Beers et al., 2006). Interestingly, down regulation of the mutant levels in microglia results in reduced disease progression (Boillée et al., 2006). In experimental autoimmune encephalomyelitis (EAE), a neurodegenerative disease model, microglial activation was inhibited by administration of ganciclovir to chimeric mice in which only the microglia express thymidine kinase that converts this drug into its cytotoxic form. Such specific microglial paralysis inhibits disease development and attenuates inflammatory CNS lesions (Heppner et al., 2005). Moreover, in Alzheimer’s disease, characterization of fibrillar plaque development in brains of transgenic APP(SW) mice revealed that microglia are not only unable to clear amyloid-β deposits, but rather, contribute to plaque formation (Stalder et al., 2001; Wegiel et al., 2001). Additionally, microglia-derived chronic inflammation was shown to precede neuronal loss in neonatal borna disease virus (BDV) infection (Ovanesov et al., 2008).

Collectively, these evident suggest that under intensive acute or chronic activation microglia fail to acquire the desired phenotype, lose their essential functions and turn actively deleterious, and thus cannot provide immune resolution and subsequent CNS protection. In such scenarios, the recruitment of additional myeloid cells from the blood, comparable to microglia, is not likely. Rather, there is a need for peripheral intervention, in the form of unique cells, capable of providing the functions that cannot be delivered by the resident microglia.

## FUNCTIONAL MACROPHAGE HETEROGENEITY

Indeed, intensive acute or chronic microglial activation drives these cells to produce a chemoattractive profile favoring the recruitment of monocytes and lymphocytes (Hausler et al., 2002; Sargsyan et al., 2009; Okamura et al., 2012). However, as the CNS is an immune privileged site, it was assumed to exclude leukocyte trafficking (Wilson et al., 2010; Ransohoff and Engelhardt, 2012). Consequently, several studies suggested that the recruitment of myeloid cells to the CNS reflected non-physiological entry, imposed by the unnatural experimental model (Ajami et al., 2007; Mildner et al., 2007). Other studies, although recognizing leukocyte entry to the CNS, interpreted their presence as a sign of pathology or malfunction that is detrimental and should be avoided (McGeer et al., 1990; Popovich et al., 1999; Gris et al., 2004; Stirling et al., 2004; Boster et al., 2008). Moreover, the previous technical limited ability to distinguish between the infiltrating blood-derived cells and the resident microglia resulted in the view of the newly recruited cells as part of the microglial reservoir, leading to their inaccurate tagging as “blood-derived microglia” (Eglitis and Mezey, 1997; Priller et al., 2001; Bechmann et al., 2005; Simard et al., 2006). This misleading nomenclature resulted in the erroneous perception of these cells as phenotypically and functionally comparable to microglia. Since then, advanced techniques have been developed to allow the blood-derived cells to be distinctly tracked and manipulated (Popovich and Hickey, 2001; Wright et al., 2001; Carson et al., 2007; Rolls et al., 2008; Shechter et al., 2009; Ajami et al., 2011; Colton, 2013). A series of recent studies used head shielded [*Cx3BR1^GFP/+^*→WT] bone marrow chimeric mice, whose wt bone marrow was replaced with that of *Cx3BR1^GFP/+^* mice (Jung et al., 2000). This approach allows the infiltrating myeloid cells, derived from donor bone marrow and labeled with GFP, to be distinguished from their resident counterparts, while avoiding any artifacts related to brain irradiation (Shechter et al., 2009). These studies revealed the unique and non-redundant functions of the newly recruited cells and suggested the term “monocyte-derived macrophages (mo-MΦ)” to identify these cells as an entity separate from the resident microglia (Shechter et al., 2009, 2011; London et al., 2011). mo-MΦ restrict amyloid-β plaques in a mouse model of Alzheimer’s disease (Simard et al., 2006; Butovsky et al., 2007), contribute to motor function recovery following spinal cord injury (Shechter et al., 2009), promote survival of neurons and cell renewal in the injured retina (London et al., 2011), and were recently shown to arrest disease progression in Rett syndrome (Derecki et al., 2012). These cells display immune-resolving characteristics and express anti-inflammatory cytokines, which are crucial for their neuroprotective function. Moreover, they restrict accumulation of other inflammatory leukocytes including neutrophils and resident microglia (Shechter et al., 2009; London et al., 2011), mediate debris clearance by phagocytosis (Derecki et al., 2012), and regulate the extracellular matrix and glial scar surrounding the damaged area (Shechter et al., 2011). Importantly, inhibition or attenuation of the infiltration of mo-MΦ results in exacerbated damage, indicating that the resident microglia, which were spared in these experiments, cannot fulfill the protective functions provided by the mo-MΦ (Butovsky et al., 2007; Shechter et al., 2009, 2011; London et al., 2011)*.*

Additional reinforcement for the disparity of these two myeloid populations is the fact that resident microglia and mo-MΦ development is dependent on different transcription factors. While development of both microglia and mo-MΦ requires the transcription factor, *Pu.1* (McKercher et al., 1996), the latter necessitates *Myb* and FLT3, whereas microglial development is *csf1*-receptor-dependent and FLT3- and *Myb*-independent (Ginhoux et al., 2010; Schulz et al., 2012; Gomez Perdiguero et al., 2013). Each of these two myeloid populations has a unique set of transcription factors and regulators leading to a diverse pattern of gene expression. Advanced analysis methods compared the profile of gene expression, microRNAs (miRNAs) and transcription factors, of splenic Ly6C^hi^ monocytes and CD39^+^ resident microglia in the SOD1 mouse, and of CD14^+^CD16^-^ peripheral monocytes in ALS patients (Butovsky et al., 2012). In this study, microglial apoptosis was demonstrated along disease progression, while the macrophages derived from Ly6C^hi^ monocytes proliferated in the spinal cord parenchyma and were associated with motor neuron loss (Butovsky et al., 2012). Notably, in this study, resident microglia were compared only to the inflammatory Ly6C^hi^ mo-MΦ population, whereas a recent study reported the recruitment of two blood-derived macrophage populations following spinal cord injury: the Ly6C^hi^CX_3_BR1^low^ pro-inflammatory and Ly6C^low^CX_3_BR1^hi^ anti-inflammatory cells, which acquire their phenotype via their trafficking route (Shechter et al., 2013). Thus, it will be interesting to characterize the gene signature of both Ly6C^hi^ and Ly6C^low^ mo-MΦ compared to resident microglia, and also to address the issue of where, how and when microglia acquire their phenotype.

All together, the evidence collected above indicates that under extensive inflammatory conditions, microglia lose their beneficial functions and instead display a deleterious role, further contributing to the spread of damage. Withstanding this vicious inflammatory cycle requires the recruitment of mo-MΦ, which induce resolution of the local immune response, rather than simply acting as microglial reinforcements. This perception leads, however, to the question of what drives the functional disparity between resident microglia and mo-MΦ. Specifically, what prevents microglia from acquiring the essential immune-resolving “alternatively activated” phenotype that is provided by the mo-MΦ? The answers to these questions may lie in the distinct origin of the two myeloid populations; microglial progenitors are yolk sac-derived macrophages that infiltrate the CNS during early embryogenesis, when bone marrow-derived hematopoiesis, from which mo-MΦ originate, is not yet established (Ginhoux et al., 2010; Schulz et al., 2012; Gomez Perdiguero et al., 2013). Educated in the CNS from early ontogeny, microglia were never exposed to any other environment; they have a relatively long life-span and undergo moderate and limited turn-over by self-renewal of primitive myeloid precursors (Ajami et al., 2007; Ginhoux et al., 2010; Schulz et al., 2012; Gomez Perdiguero et al., 2013). In contrast, mo-MΦ are freshly recruited from the circulation. Their differences may also be related to the fact that microglia are the first to encounter the damaged tissue, which might dictate their phenotype, while the mo-MΦ, recruited at a slightly later stage, acquire their nature via their trafficking route to the CNS (Shechter et al., 2013). Interestingly, such timely recruitment of myeloid subsets with differential functions is also evident in insults of non-CNS organs (Arnold et al., 2007; Nahrendorf et al., 2007). Clearly, further research is needed in order to address these issues.

## FUNCTIONAL RELATIONSHIPS BETWEEN THE MICROGLIA AND mo-MΦ – A CASCADE OF EVENTS

**FIGURE 1:**
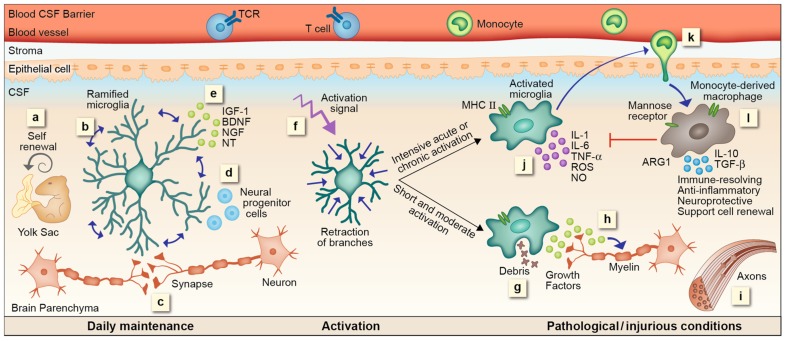
**microglial and mo-MΦ functions – cascade of events**. (a) Resident microglia originate from yolk sac macrophages that repopulate CNS parenchyma during early development and are self-renewed locally, independent from bone marrow-derived monocytes, by proliferation of primitive progenitors. (b) In the steady state microglia are constantly scanning their environment through their highly motile processes. These cells facilitate the maintenance of synapses (c) and neurogenesis (d), as well as secrete growth factors essential for normal CNS performance (e). Upon recognition of a danger signal, microglia retract their branches, become round and ameboid, and convert into an activated mode (f). A short or moderate signal directs microglia toward a neuroprotective phenotype; these cells clear debris by phagocytosis (g), secrete growth factors associated with remyelination (h) and support regeneration (i). Intensive acute or chronic activation renders microglia neurotoxic; under such conditions microglia fail to acquire a neuroprotective phenotype. Instead, these cells produce reactive oxygen species (ROS), nitric oxide (NO), proteases, and pro-inflammatory cytokines such as IL-1, IL-6, and TNF-α, all of which endanger neuronal activity (j). Microglial malfunction results in the recruitment of mo-MΦ to the damage site (k). mo-MΦ secrete anti-inflammatory cytokines such as IL-10 and TGF-β, express factors associated with immune resolution such as manose receptor and arginase 1 (ARG1), and promote neuroprotection and cell renewal (l), all of which are functions that cannot be provided, under these conditions, by the resident microglia.

Based on the data reviewed above, we suggest here a cascade of events representing microglial functions within the CNS and the distinct contribution of mo-MΦ. Microglia enter the CNS during early developmental stages. By continuous scanning and sampling their environment via their dynamic processes, microglia are able to maintain CNS homeostasis; they preserve and modify (upon need) the synapse complex, support neurogenesis, secrete essential growth factors, and sustain normal CNS performance. Once encountering an unbalanced milieu, microglia become fully activated; retract their long ramifications, proliferate and shift toward a “ready to act” mode. Their subsequent function is very much dependent on their activation signal. A short and moderate stimulus will direct microglia to rapidly eliminate the source of damage without evoking a further immune response. Such stimuli are part of routine CNS maintenance and are generally resolved without activating or affecting other systems in the body. Even when the stimulus is stronger but short-lived, microglia can potentially cope with the danger signal, performing clearance of neurotoxic factors, supporting regeneration, and secreting neurotrophic factors supportive of remyelination. However, when the stimulus is intense or chronic, microglia can no longer handle the ongoing damage; these cells become neurotoxic and release reactive oxygen species, NO, proteases and pro-inflammatory cytokines, all of which endanger neuronal activity. Such microglial malfunction results in signals for recruitment of mo-MΦ to the damage site, which provide functions that cannot be delivered by the resident cells; mo-MΦ restrict the local inflammation, attenuate accumulation of misfolded proteins or any other intruders, restore homeostasis, and support healing and renewal. Unfortunately, the spontaneous response of mo-MΦ is often insufficient to achieve complete recovery. Thus, several therapeutic attempts to boost such a protective response by either direct administration of monocytes or indirectly augmenting their recruitment are currently underway (**Figure 1**).

## LESSONS FROM OTHER TISSUE-SPECIFIC RESIDENT MACROPHAGES

**FIGURE 2:**
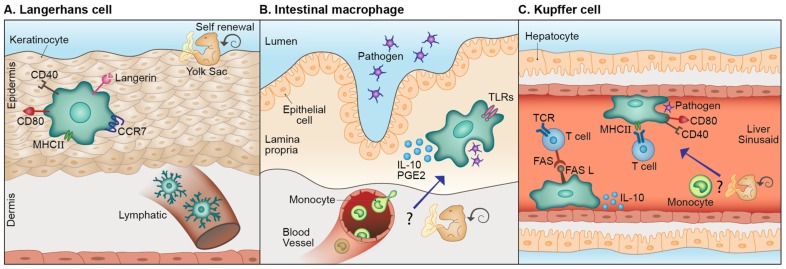
**Lessons from other tissue-specific resident macrophages**. **(A)** Langerhans cells (LCs), the resident myeloid cells of the epidermis share with microglia their scanning capacity, their activation mode and possibly, their embryonic origin. Different from microglia these cells migrate to the lymph node where they act as antigen presenting cells. **(B) **Analogous to microglia, intestinal macrophages act as the first line of defense, protecting the mucosa from harmful pathogens and removing dead cells and debris. Unlike most other tissue macrophages, upon activation by certain stimuli, these cells produce immune-resolving factors. Distinct from microglia, circulating monocytes are largely accepted as the source of intestinal macrophages, however, the possibility of local self-renewal by embryonic precursors, under steady state, was also raised.** (C)** Kupffer cells are the macrophages of the liver. Similar to microglia, these cells perform clearance of host-related debris and pathogens. Kupffer cells are classical antigen presenting cells; however can also display immune-resolving functions. Moreover, they are largely assumed to be self-renewed independently from circulating monocytes, but a certain Kupffer cell subset was reported to originate from hematogenous precursors.

Although unique, microglia are not the sole tissue-specific resident myeloid-derived cells. Many organs in the body contain distinctive resident macrophages whose properties are tailored to the host tissue (**Figure 2**). Langerhans cells (LCs), for example, are the resident myeloid cells of the epidermis. Similar to microglia, they have extended dendritic processes that embrace neighboring keratinocytes (Langerhans, 1868; Bilzer et al., 2006) and scan the epidermis for pathogens and toxic molecules (de Jong and Geijtenbeek, 2010). These cells are endowed with the C-type lectin, Langerin, used for interaction with bacteria, fungi, and viruses (Turville et al., 2002; de Witte et al., 2007; Merad et al., 2008; de Jong and Geijtenbeek, 2010). Like microglia, LCs descend from embryonic precursors, possibly yolk sac macrophages or fetal liver monocytes, and are renewed independently of the bone marrow, by *in situ* proliferation upon need (Merad et al., 2002, 2008; Chorro et al., 2009; Chorro and Geissmann, 2010; Hoeffel et al., 2012). Moreover, as in microglial activation, upon capture of pathogens, LCs undergo phenotypic changes including increased expression of MHC-I and -II, and of the co-stimulatory molecules CD80, CD86, and CD40 (Merad et al., 2008; de Jong and Geijtenbeek, 2010). However, unlike microglia, which are restricted to the CNS parenchyma, LCs upregulate the lymph node-homing receptor, CCR7, which eventually leads to their migration to peripheral lymph nodes where they induce a specific adaptive immune response against skin invading pathogens (Merad et al., 2008). Intestinal macrophages are the largest population of mononuclear phagocytes in the body (Smith et al., 2005; Varol et al., 2010; Mowat and Bain, 2011). Similar to microglia, they have essential functions under both normal and pathological conditions; intestinal macrophages preserve a delicate equilibrium between commensal bacteria and the host, maintaining epithelial integrity and mucosal homeostasis. These cells act as the first line of defense protecting the highly exposed mucosa from harmful pathogens, removing dead cells and debris, and modulating the local inflammatory response (Smith et al., 2005; Varol et al., 2010; Mowat and Bain, 2011). Unlike other tissue macrophages, upon activation, for instance by certain Toll-like receptor (TLR) ligands, intestinal macrophages do not express high levels of co-stimulatory molecules nor do they secrete pro-inflammatory cytokines (Rogler et al., 1998; Hirotani et al., 2005; Uematsu et al., 2006; Mowat and Bain, 2011; Smith et al., 2011). Rather, they produce anti-inflammatory mediators such as IL-10 and prostaglandin E2 that restrict the local immune response (Mowat and Bain, 2011). Unlike microglia, the replenishment of intestinal macrophages is mostly associated with the recruitment of circulating monocytes. However, the possibility of self-renewal under steady state has also been raised (Mowat and Bain, 2011). Kupffer cells are the macrophages of the liver. These cells are mainly involved in clearance of pathogens and host-derived waste; they are constantly exposed to bacterial endotoxin (LPS) and microbial debris delivered from the gastrointestinal tract (Naito et al., 2004) and are involved in removal of senescent or malformed red blood cells and phagocytosis of soluble immunoglobulin G (IgG) complexes, microorganisms and eukaryotic cells (Naito et al., 2004; Parker and Picut, 2012). In addition to their role as phagocytes, Kupffer cells act as effective antigen presenting cells; upon Hepatitis C virus infection, human Kupffer cells elevate MHC-I and -II expression, upregulate co-stimulatory molecules, and interact with T cells (Burgio et al., 1998). However, several studies also demonstrated the immune-resolving nature of Kupffer cells, which were shown to suppress lymphocytes in culture (Callery et al., 1991), secrete IL-10 in response to LPS challenge (Knolle et al., 1995) and facilitate Fas ligand (FasL)-mediated apoptosis of T cells in a liver transplant model (Miyagawa-Hayashino et al., 2007). Thus, similarly to CNS heterogeneous macrophages, Kupffer cells seem to perform highly versatile functions. These cells, like microglia and LCs appear to self-renew independently of bone marrow-derived precursors (Schulz et al., 2012; Gomez Perdiguero et al., 2013). However, a study addressing Kupffer cell heterogeneity identified two subsets of Kupffer cells; one of them is radiosensitive and rapidly replaced from hematogenous precursors (Klein et al., 2007), indicating that the issue of Kupffer cell renewal is still unresolved.

## CONCLUSIONS AND FUTURE DIRECTIONS

The evidence collected in this perspective supports the concept of functional macrophage heterogeneity within the CNS. Due to their similar morphology, previously assumed shared origin and subsequent misleading nomenclature, as well as the lack of available techniques to distinguish between the two populations, microglia and mo-MΦ were erroneously assumed to comprise a single population. Alternatively, and based on the ample findings addressed above, our model suggests that when it comes to CNS macrophages initial impressions can be deceiving; although they appear similar, mo-MΦ and microglia present different gene expression patterns and phenotypes, and are functionally distinct. Additional research is needed in order to further reveal the different function of these two distinct populations and the conditions that determine their unique phenotype. Such research will help resolving the misunderstanding that resulted from the previously held blanket view of these cells as homogeneously destructive, and might assist in employing specific manipulations of the two subsets as a potential therapeutic approach.

## Conflict of Interest Statement

The authors declare that the research was conducted in the absence of any commercial or financial relationships that could be construed as a potential conflict of interest.
